# Supplementary material for: Dengue in Western Uganda: a prospective cohort of children presenting with undifferentiated febrile illness
Source: BMC Infect Dis. 2020 Nov 11;20:835. doi: 10.1186/s12879-020-05568-5 (PMC7661220; doi:10.1186/s12879-020-05568-5)
Supplement: Supplementary file 1 — Additional file 1. [file 12879_2020_5568_MOESM1_ESM.zip › DNMFI - Encounter Form.doc]

| **Study Identification Number (**Site **-** Day – Month - Number): --- --- --- | | | | | | | | |
| --- | --- | --- | --- | --- | --- | --- | --- | --- |
| **SECTION 1: PATIENT INFORMATION** | | | | | | | | |
| **Demographics:** | **Sex:** | | **Age:** | | | | | |
|  | 🞎 Male | | Years: | | | | | |
|  | 🞎 Female | | Months (for children <2 years only): | | | | | |
| **SECTION 2: HISTORY & PRESENT ILLNESS** | | | | | | | | |
| **Symptoms:**  🞎 skin rash 🞎 fever  🞎 cough 🞎 fatigue  🞎 headache 🞎 sore throat  🞎 runny nose 🞎 body/joint pains  🞎 diarrhea 🞎 vomiting  🞎 not eating 🞎 painful urination  🞎 convulsions 🞎 bleeding  🞎 coma 🞎 jaundice  🞎 Other: ____________________ | | **Clinical History:**  Days since first fever: _______  Seen at another Health Centre?  🞎 Yes  🞎 No  Taken drug for malaria?  🞎 Yes  🞎 No  Traditional treatment?  🞎 Yes ____________  🞎 No | | | **Past Medical History:**  🞎 Asthma  🞎 Diabetes  🞎 Epilepsy  🞎 Heart problems  🞎 High blood pressure  🞎 ISS  🞎 Malaria  🞎 Pregnant  🞎 Tuberculosis  **Other conditions:** | | | |
|  |  |  |  |  |  |  |  |  |
|  |  |  |  |  |  |  |  |  |
|  |  |  |  |  |  |  |  |  |
|  |  |  |  |  |  |  |  |  |
|  |  |  |  |  |  |  |  |  |
|  |  |  |  |  |  |  |  |  |
|  |  |  |  |  |  |  |  |  |
|  |  |  |  |  |  |  |  |  |
|  |  |  |  |  |  |  |  |  |
|  |  |  |  |  |  |  |  |  |
|  |  |  |  |  |  |  |  |  |
|  |  |  |  |  |  |  |  |  |
|  |  |  |  |  |  |  |  |  |
| **SECTION 3: OBJECTIVE DATA** | | | | | | | | |
| **Current Temperature:** | | | **Malaria Test Type:** 🞎 RDT 🞎 Microscopy | | | | | |
|  |  |  | **Malaria Test Result:** 🞎 POS 🞎 NEG | | | | | |
| **SECTION 4: CONFIRM INCLUSION CRITERIA** | | | | | | | | |
| **Does patient have:** | 🞎 **History of fever or current fever**  **AND:**  🞎 Negative malaria test (RDT or microscopy) | | *If the patient meets all three criteria, then they are eligible for the study. Please perform a Dengue RDT and collect the appropriate blood samples.* | | | | | |
| **SECTION 5: RESULTS** | | | | | | | | |
| **DENGUE RDT – Check all that apply** | | 🞎 Negative | | 🞎 NS1 POS | | 🞎 IgM POS | | 🞎 IgG POS |
| **SECTION 6: PATIENT DISPOSITION** | | | | | | | | |
| **Status** | 🞎 Received antimalarial (Co-artem) | | 🞎 Received antibiotics | | | | 🞎 Admitted | |
|  | 🞎 Other (specify): | | | | | | | |
